# Using response-time latencies to measure athletes’ doping attitudes: the brief implicit attitude test identifies substance abuse in bodybuilders

**DOI:** 10.1186/1747-597X-9-36

**Published:** 2014-09-10

**Authors:** Ralf Brand, Wanja Wolff, Detlef Thieme

**Affiliations:** 1 Department of Sport and Exercise Psychology, University of Potsdam, Am Neuen Palais 10, 14469 Potsdam, Germany; 2 Institute of Doping Analysis and Sports Biochemistry Dresden, WADA (World Anti-Doping Agency) accredited doping control laboratory, Dresdner Str. 12, 01731 Kreischa, Germany

**Keywords:** Steroid use, Psychology, Doping tests, Biochemical profiles

## Abstract

**Background:**

Knowing and, if necessary, altering competitive athletes’ real attitudes towards the use of banned performance-enhancing substances is an important goal of worldwide doping prevention efforts. However athletes will not always be willing to reporting their real opinions. Reaction time-based attitude tests help conceal the ultimate goal of measurement from the participant and impede strategic answering. This study investigated how well a reaction time-based attitude test discriminated between athletes who were doping and those who were not. We investigated whether athletes whose urine samples were positive for at least one banned substance (dopers) evaluated doping more favorably than clean athletes (non-dopers).

**Methods:**

We approached a group of 61 male competitive bodybuilders and collected urine samples for biochemical testing. The pictorial doping Brief Implicit Association Test (BIAT) was used for attitude measurement. This test quantifies the difference in response latencies (in milliseconds) to stimuli representing related concepts (i.e. doping–dislike/like–[health food]).

**Results:**

Prohibited substances were found in 43% of all tested urine samples. Dopers had more lenient attitudes to doping than non-dopers (Hedges’s *g* = -0.76). *D*-scores greater than -0.57 (CI95 = -0.72 to -0.46) might be indicative of a rather lenient attitude to doping. In urine samples evidence of administration of combinations of substances, complementary administration of substances to treat side effects and use of stimulants to promote loss of body fat was common.

**Conclusion:**

This study demonstrates that athletes’ attitudes to doping can be assessed indirectly with a reaction time-based test, and that their attitudes are related to their behavior. Although bodybuilders may be more willing to reveal their attitude to doping than other athletes, these results still provide evidence that the pictorial doping BIAT may be useful in athletes from other sports, perhaps as a complementary measure in evaluations of the effectiveness of doping prevention interventions.

## Background

Doping is defined as the presence of a prohibited substance or its metabolites or markers (including elevated quantities of endogenous substances), or evidence of use of a prohibited method, in an athlete’s sample. Although only 1.19% of the 267,645 samples analyzed and reported to the World Anti-Doping Agency (WADA) by accredited laboratories in the year 2012 produced adverse analytical findings [1], the percentage of doping cases which remain unrevealed is thought to be considerably higher. For example, recent analyses of biochemical data from 2,737 elite track and field athletes exposed evidence of blood doping in an average of 14% of athletes, with prevalence varying considerably across nationalities (between 1% and 48% in range) [2].

There is strong evidence that attitudes can be used to predict behavior [3]. Several studies have already reported correlations between attitudes to doping, social cognitive determinants of doping behavior and self-admitted doping. For example, a study reported that attitude to doping was a significant predictor of doping intention in a sample of 1,075 Greek elite athletes [4]. Another study of a sample of 729 athletes reported that attitudes to doping helped to explain participants’ willingness to dope [5]. Another study used data from 750 elite athletes to demonstrate that sportspersonship orientations and achievement goals exerted an indirect influence on doping intentions through their influence on attitudes to doping and self-efficacy beliefs [6]. A recent meta-analysis concluded that attitudes to doping are amongst the strongest predictors of self-admitted doping behavior [7].

Extant psychological studies on doping behavior rely almost exclusively on verbal self-reports of attitudes to doping and use of doping. Unfortunately direct self-report instruments, for example questionnaires on doping-related cognition or use of doping, are prone to a social desirability bias [8]. Athletes will not always report their attitude or behavior frankly, they may start to deliberate and give what they believe to be the socially desirable response [9].

The Implicit Association Test (IAT) is a reaction time-based method which helps to address the problem of strategic answering in such situations [10-12]. There is ample evidence that IAT effects can be interpreted as indicators of attitudes [13].

The IAT is based on the theoretical assumption that knowledge is stored in our memory in an association network. Information is stored as nodes in an associative network of semantic information. If a node is activated the activation spreads through the network and associated nodes are automatically activated [14]. Activation of a doping-related semantic concept, for example by presenting the words *Erythropoietin or EPO* (a drug which can be used as a doping substance), should automatically activate the associated evaluation (e.g. *positive* or *negative*).

The test is typically presented on a computer in the form of a lexical sorting task in which two concepts (one target and one evaluative) are mapped to the same computer key. The sorting task is easier for the respondent (and therefore completed more quickly) when the two concepts sharing the same response key are closely associated than when they are not (e.g. an athlete who disapproves of doping will respond more quickly when *doping* and *dislike* are mapped to the same key than when *doping* and *like* are mapped to the same key). IAT scores are calculated by using the difference between response times for related and unrelated pairs. Response times are interpreted as a measure of the associative strength between the target concept and the attribute.

To date relatively little research has directly addressed the associative and automatic nature of the process underlying the IAT effect [13]. Non-associative, e.g. propositional accounts therefore remain viable alternative interpretations of IAT effects [15,16] although at present it seems unreasonable to reject the assumption of an underlying associative process [17]. The IAT is currently the method of choice for testing automatic associations, as it incorporates core features of implicit psychological measurement [18].

Petróczi, Aidman and Nepusz reported seminal work on use of IATs for measuring attitudes to doping [19]. They showed that subjects responded faster to pairings of *doping* with *bad* than to pairings of *doping* with *good*. Brand, Melzer and Hagemann [20] compared the measurement properties of this early version of a doping IAT [19] with an alternative procedure [21] and showed that test results were strongly dependent on the test stimuli used. Shortly thereafter Petróczi et al. [22] published preliminary results from a study in which a brief IAT (BIAT) was employed. The BIAT is a shorter version of the standard IAT procedure [23]. Six athletes who denied current doping in a questionnaire although biochemical traces of banned substances were detected in their hair samples responded more rapidly to the *good*-*doping* pair than the 4 athletes who admitted doping. However the difference was very small and the variance was large so the result was not statistically significant. The authors elaborated on these preliminary findings in a later article [24], concluding that self-reported attitudes to doping in a questionnaire aligned well with admitted use, whereas BIAT results aligned better with biochemical test results (although differences in response latencies did not reach statistical significance). Their results suggested that an exaggerated divergence between direct (questionnaire self-report) and indirect (BIAT) measures of attitude might be characteristic of deniers, whilst clean athletes would show convergent response patterns. There were limitations to these two studies [22,24], namely that the findings were based on a sample of only 10 athletes and the method of biochemical testing was suboptimal (e.g. there was no differentiation between endogenous hormones and introduced substances).

Recently Brand, Heck and Ziegler [25] introduced a new BIAT for measuring athletes’ attitudes to doping that uses pictures instead of word stimuli. This test might thus provide the basis for a transnationally valid instrument which could be used as part of a harmonized worldwide anti-doping effort. In their study the new doping BIAT was found to have adequate psychometric properties and athletes’ attitudes to doping were shown to be associated with how prone their discipline was to doping.

In order to investigate whether this pictorial doping BIAT [25] successfully discriminates dopers from non-dopers, we investigated a sport in which the probability of doping is thought to be particularly high: bodybuilding is not subject to WADA regulations, and use of anabolic steroids is seen as an integral part of the culture of the sport by most bodybuilders [26]. Although compelling evidence from epidemiological studies is very rare, there has been at least one previous study in which between 38 and 58% of competitive bodybuilders had positive biochemical test results indicating the use of performance enhancing drugs (i.e. doping in most other sports) [27].

This study extends the current knowledge as it tested the hypothesis that lenient attitudes to doping are linked with doping behavior. Results of biochemical urine analyses were used to classify participating bodybuilders as dopers or non-dopers, and biochemical profiles of doping-users in bodybuilding are given. The pictorial doping BIAT [25] was used to measure participants’ attitudes to doping. We assessed the sensitivity and specificity of the pictorial doping BIAT as a means of discriminating dopers and non-dopers, hypothesizing that dopers would have a more lenient attitude to doping.

## Materials and methods

### Participants and data collection

On the basis of the result of an *a priori* power calculation (*n* ≥ 52; independent samples t-test; Cohen’s *d* = 0.7, α = 0.05 and 1-β = 0.80), we sampled 61 male competitive bodybuilders (*M* = 30.36 ± 9.29 years old). These bodybuilders were a convenience sample. All data were collected between June and October 2013. Two research assistants recruited bodybuilders personally during training at their home gyms. They were asked directly if they would be willing to participate in a confidential research study which would involve them providing information about their age, education, training time per week and competitive experience in a paper-pencil questionnaire and then working through a computer-based test that would reveal their attitude to doping and, finally, providing a doping control urine sample. All biochemical samples were treated as in-competition doping tests and analyzed in one of Germany’s WADA-accredited laboratories (Institute of Doping Analysis and Sports Biochemistry, Dresden) following procedures laid out in the WADA International Standard for Laboratories [28]. Testing started after participants had provided informed consent in accordance with the World Medical Association’s Declaration of Helsinki (October 2008). Ethical approval for the study was obtained by the University of Potsdam on application number 39/2011.

### Reaction time-based attitude test

A recent publication provided all the information required to use the pictorial doping BIAT, including test specifications and psychometric properties [25]. The pictorial doping BIAT begins with a training block (20 trials), in which participants learn to discriminate between the focal *doping* and the non-focal *health food* pictures. After this training block participants perform one *doping*-*like* block (20 trials) and one *doping*-*dislike* block (20 trials). Block presentation order is randomized across participants to avoid position effects. *D*-scores are calculated according to the improved scoring algorithm [29] such that more positive scores indicate a more lenient attitude to doping. Error rates in this study were 17.8% (*SD* = 16.9) in the *doping*-*like* blocks and 8.9% (*SD* = 9.3) in the *doping*-*dislike* blocks.

The test was programmed using the Inquisit 3.0™ software. It was run on a custom 15.6″ computer notebook (MS Windows 7; Intel Core i5 2410 M, 2.3 GHz) with a QWERTZ keyboard.

### Biochemical tests

Urine samples were analyzed using mass spectrometry in combination with either liquid or gas chromatography and immunoassay was used to detect human chorionic gonadotropin (hCG). All tests were carried out according to WADA regulations and technical documents defining relevant prohibited substances [30], eligible analytical methodology and identification criteria [31], detection thresholds [32], decision thresholds, sample processing and reporting of results [28]. The analytical strategies for all the prohibited substance classes we investigated, including testing procedures for detection of anabolic agents, peptide hormones, stimulants and hormone and metabolic modulators are described exhaustively in the literature [33]. Doping substances were classified according to the WADA 2013 list of prohibited substances [30].

## Results

### Biochemical tests

Biochemical analyses revealed that 42.6% (26 out of 61) of the bodybuilders in our sample used at least one prohibited substance at the time of testing. We found metabolic modulators, stimulants, cannabinoids (in one athlete’s sample), the anabolic β2-agonist clenbuterol, and a broad range of anabolic steroids (Table 1). The anabolic agents found were testosterone, several conventional synthetic analogues (e.g. drostanolone, trenbolone, stanozolol) and methasterone (in one athlete’s sample). This last substance was recently introduced onto the black market as a designer steroid [34]. Cases of severe liver damage due to this substance are documented in the literature [35]. The testosterone-epitestosterone (T/E) ratio was >10 in 75% of all doping-positive samples (a ratio >4 is usually taken as an indication of administration of exogenous testosterone). Carbon isotope ratio mass spectrometry [36] was used to confirm exogenous origin where necessary, i.e. in cases where testosterone levels were the only evidence of doping. Only three of our doping-positive bodybuilders did not use anabolic agents; nine used only one anabolic agent; two anabolic substances were found in four samples; six in three samples; one participant used four (boldenone, drostanolone, nandrolone, trenbolone), and another six anabolic agents (boldenone, drostanolone, metenolone, nandrolone, stanozolol, trenbolone). A table providing complete biochemical test results for all individual participants is provided (Additional file 1: Table S1).

**Table 1:** Prohibited substances in urine samples of bodybuilders (n = number of participants)

**S1**	**S2**	**S4**	**S6**	**S8**
**Anabolic agents**	**Peptide hormones, growth factors and related substances**	**Hormone and metabolic modulators**	**Stimulants**	**Cannabinoids**
Methandienone (n = 5)	hCG (n = 1)	Tamoxifene (n = 6)	Methylhexanamine (n = 2)	cTHC (n = 2)
Boldenone (n = 13)		Anastrozol (n = 2)	Amphetamine (n = 1)	
Drostanolone (n = 4)			Ephedrine (n = 1)	
Methasterone (n = 1)			MDMA (n = 1)	
Metenolone (n = 1)				
Nandrolone (n = 11)				
Oxymetholone (n = 1)				
Stanozolol (n = 4)				
Testosterone (n = 20)				
Trenbolone (n = 6)				
Clenbuterol (n = 1)				

Substances are grouped (S1 to S8) according to the WADA Prohibited List 2013.^15^ hCG (human chorionic gonadotropin) induces the endogenous biosynthesis of testosterone, MDMA (3,4-methylenedioxyamphetamine) is the stimulating entactogen marketed as ecstasy, and cTHC (11-nor-9-carboxy-THC) is the urinary marker of THC-administration.

### BIAT and biochemical test results

**Figure 1:**
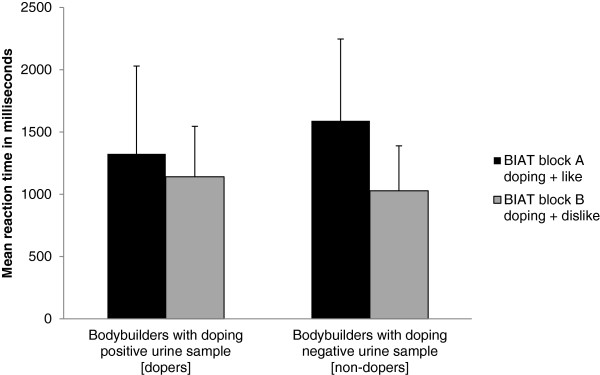
**Results from the doping BIAT attitude measurement.** Bars show mean reaction times (±1 standard deviation) in groups of bodybuilders whose urine samples either contained (dopers; n = 26) or did not contain (non-dopers; n = 35) forbidden substances according to the WADA prohibited list 2013. Block A refers to the BIAT’s part, in which the target concept *doping* and the evaluative concept *like* are mapped on the same response key (and block B respectively).

Bodybuilders whose urine samples contained a prohibited substance (*n* = 26) did not differ in age, education, weekly training minutes and years of competitive experience from those who tested negative (*n* = 35; all *p* ≥ .13, all Hedges’s *g* ≤ 0.32). Mean BIAT response latencies for dopers and non-dopers are depicted in Figure 1. The mean *D*-score for the group of non-dopers (*M* = -0.52, *SD* = 0.50) was significantly more negative than for the group of dopers (*M* = -0.12, *SD* = 0.50); *t*(59) = -2.95, *p* = .002, Hedges’s *g* = -0.76 (CI95 = 0.24 to 1.29), indicating that dopers’ reactions were faster than non-dopers’ when the BIAT target concept *doping* and the evaluative category *like* were mapped to the same response key, which suggests that the two concepts were more closely associated for dopers than for non-dopers. As a group the dopers in our sample showed more lenient attitudes to doping than the non-dopers. Individual *D*-scores for all the bodybuilders are included in the table given as Additional file 1: Table S1.

**Figure 2:**
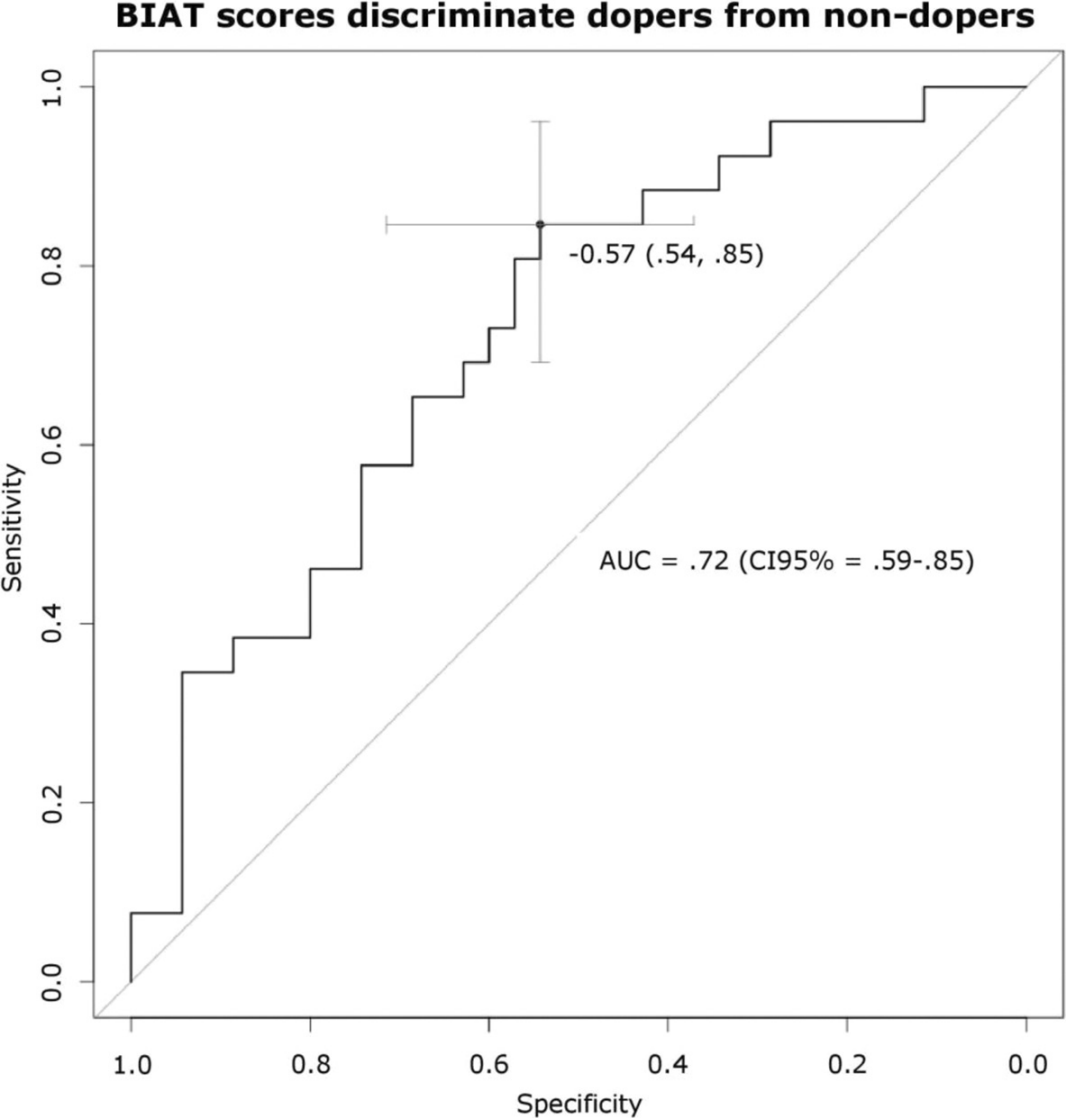
**Results of ROC analysis (receiver operating characteristic).** The ROC curve (black) describes the BIAT’s performance in discriminating dopers from non-dopers. The ROC graph depicts relative tradeoffs between true positives (test sensitivity) and false positives (test specificity) as a function of each obtained BIAT score. The farther the AUC (Area Under the Curve = 0.722 ± CI95%) deviates from the diagonal (grey) representing chance level (AUC = 0.50), the better the BIAT’s performance is in terms of maximizing true positive and true negative cases. At the proposed BIAT cut-off score (-0.57), specificity (54.3%) and sensitivity (84.6%) are maximized.

Results from a subsequent receiver operating characteristic (ROC) analysis (Figure 2) revealed that BIAT *D*-scores could be used to discriminate dopers from non-dopers. The overall accuracy of the test can be quantified in terms of the area under the curve (AUC), which in this sample was .72, indicating that dopers have higher (more positive) BIAT scores than 72% of the non-dopers. The threshold BIAT *D*-score which maximized test sensitivity and specificity was -0.57 (CI95 = -0.72 to -0.46).

**Figure 3:**
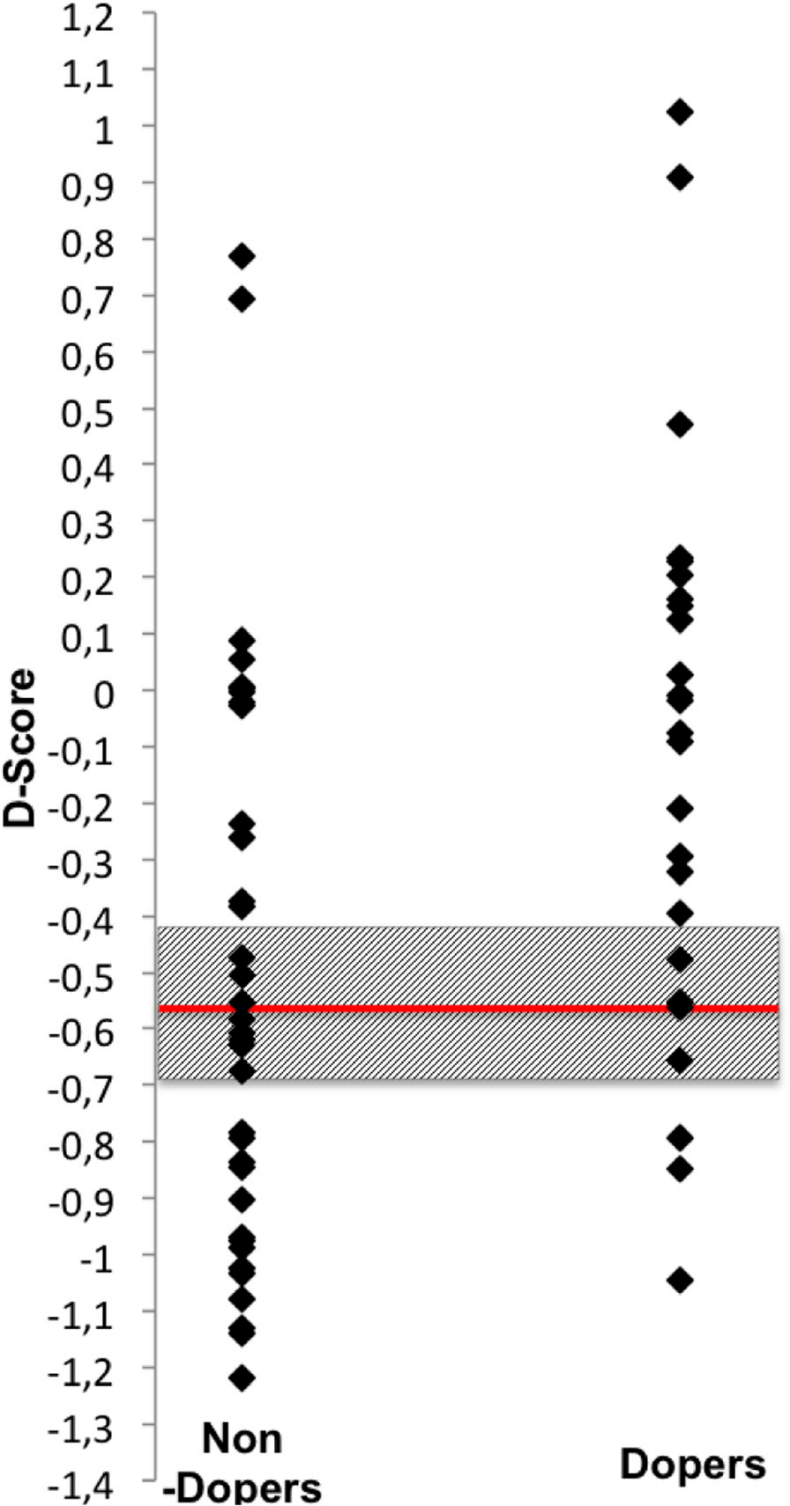
**Application of proposed reference thresholds on doping BIAT *D*-scores.** Dots represent all bodybuilders’ (dopers and non-dopers) individual *D*-scores. Lower and upper limits of the grey box represent the range of the 95% confidence interval (-0.72, -0.46) around the cut-off *D*-score (horizontal line at -0.57) as proposed by the ROC analysis.

When applied to individual bodybuilders in our sample (Table 2) this threshold leaves 15.38% of all dopers undetected (false negatives), and inadvertently classifies 45.71% of clean bodybuilders as dopers (false positives). The threshold *D*-score or the upper and lower limits of the 95% confidence interval might be useful as reference scores in other studies that use the pictorial doping BIAT. An illustration of *D*-score distributions in our groups of dopers and non-dopers together with the proposed threshold reference scores is given in Figure 3.

**Table 2:** **True hits and false alarms (number of participants) produced by the doping BIAT cut-off *D*-score, proposed to discriminate test participants (here: bodybuilders) with a rather negative (*D*** **≤ -0.57) or a rather positive (*D*** **> -0.57) doping attitude**

		**BIAT: doping attitude**	
		**Rather negative evaluation of doping**	**Rather positive evaluation of doping**
Biochemical test: presence of doping substance in urine sample	Doper	4	22
	Non-doper	19	16

## Discussion

The pictorial doping BIAT [25] revealed that bodybuilders whose urine sample contained at least one banned substance had more lenient attitudes to doping than clean bodybuilders. This reaction time-based indirect measure of attitude can be helpful as a complementary indicator of athletes’ attitudes to doping. Test scores could for example be used to inform decisions about whether or not athletes should be recommended for doping prevention interventions. The diagnostic accuracy of the proposed reference scores is important in the context of potential practical applications, as is the more general question of whether findings in bodybuilders will generalize to other sports.

One of our main claims is that these findings based on a sample of bodybuilding athletes will generalize to other sports (e.g. Olympic sports such as track and field athletics, swimming, handball and association football). Unlike in these other sports, in bodybuilding the use of performance-enhancing drugs (primarily anabolic steroids) is neither formally nor socially prohibited. It is therefore possible that bodybuilders have a different attitude and approach to doping and perhaps even report their thoughts, feelings and behaviors with respect to doping more honestly [37]. In a very small sample Petróczi et al. [24] found that dopers could best be discriminated by the dissociation between direct and indirect indicators of attitudes to doping (dopers reported strong rejection of doping in self-report questionnaires, but reaction time-based tests indicated a relatively positive attitude). In our study the indirect test alone was sufficient to identify dopers. The dissociation pattern may only be an indicator of doping in sports where doping is prohibited, which is not the case in bodybuilding.

The results of a previous study were consistent with this hypothesis, direct and indirect measures of attitude were positively correlated in bodybuilding but uncorrelated in handball [25]. This is the reason why we did not include a direct measure of attitude in this investigation. The dissociation hypothesis remains an important research issue and theoretical accounts of the effects should be developed.

Although bodybuilders may be characterized by more lenient attitudes to doping than other athletes their scores on the pictorial BIAT may well be comparable to those of athletes from other sports. In the only study to date in which the pictorial doping BIAT was used with athletes from an Olympic sport [25] 21 bodybuilders attitudes to doping were compared with those of 22 handball players (handball was chosen because it is one of the sports in which doping is supposed to be less prevalent). The average *D*-score in the bodybuilder group was -0.14, which is very close to the -0.12 for doped bodybuilders in this study (suggesting that the majority of bodybuilders in the previous study were dopers). The mean *D-*score for the handball player group was -0.40, similar to the -0.52 score for clean bodybuilders in this study. In our view this corroborates the validity of the pictorial BIAT as an indirect measure of attitude to doping in a range of competitive sports, but additional evidence is needed to confirm this.

Bodybuilders may be less likely to fake a more negative doping attitude than athletes from other sports. It is important to note that IAT results can be faked to a certain degree under specific circumstances [38]. From an ethical point of view it is important to inform participants that the BIAT is used to assess attitudes to doping before they take the test, and this is the procedure we followed in this study. There is currently no evidence on faking in BIAT tests, but it is sensible to assume that the pictorial doping BIAT can be faked in a similar fashion to the IAT. Research addressing the issue of faking is urgently needed, as attempts to fake the test will undoubtedly affect its validity and reliability and – not least important – the applicability of the proposed reference scores (see discussion below). We can only speculate about our participants’ high compliancy with testing or motivation to fake. Regardless of the level of attempted and successful faking, and more importantly, there is little reason to suspect that there are fundamental differences between bodybuilders and athletes from other sports in terms of the general processes of attitude formation and the ways attitudes in which can be assessed. We therefore propose that the test validation data from this study should be accepted as a provisional indication that the pictorial doping BIAT is also valid for athletes from Olympic sports (i.e. other sports than bodybuilding).

Our statistical analyses suggested that pictorial doping BIAT *D*-scores greater than -0.57 were associated with a rather lenient attitude to doping and that non-dopers are more likely to have scores below this threshold than dopers. In our sample this threshold correctly identified 84.6% of dopers; however it also had a high false positive rate, 45.7% of athletes were mistakenly classified as dopers.

In our view it is reasonable to accept the surplus sensitivity of this threshold. We base our view on the pictorial doping BIAT’s potential as a complementary measure of and the paramount importance of doping prevention. We think that it is more important to identify athletes with comparatively lenient attitudes to doping in order that they can be recommended for preventative interventions than to avoid recommending athletes for such measures when they already have a negative attitude to doping. It has been argued that inappropriate use of prevention interventions could produce a boomerang effect [39]. These authors suggested that athletes who have never previously considered doping, might be prompted to do so by attending a doping prevention program [40]. We feel that this argument is only valid for athletes who would have not been faced with doping either way. This is very unlikely, at least in athletes with a background in Olympic sport who compete at an advanced level. Nonetheless researchers or organizations who use the pictorial BIAT e.g. as a complementary measure to screen for eligibility for doping prevention interventions should bear in mind that the possibility of a boomerang effect cannot be ruled out at present.

It is important to note that all groups that have been investigated with the pictorial doping BIAT so far ([25]; this study), had a mean score above the proposed threshold doping BIAT *D*-score = -0.57. On the one hand this might indicate that all the groups investigated so far have relatively positive attitudes to doping, but then it might also be an artifact due to the sensitivity-specificity trade-off discussed above. In the context of this study it is important to consider a third explanation for the relatively high *D-*scores and a caveat which applies to the interpretation of absolute *D*-scores more generally.

Biochemical testing of urine samples provides a relatively short detection window, varying from only several hours up to a few days depending on factors such as the substance used, the method and duration of use, sample storage condition etc. [41]. It is therefore possible that negative biochemical results in this study were obtained from dopers who were ‘off-cycle’ at the time of data collection (i.e. taking a break from administration of doping substances). This means that we cannot rule out the possibility that bodybuilders with a long history of doping were assigned to the non-doper group. If this were the case it might explain the relatively large number of false positives (see above) and thus strengthen our case for relying on BIAT scores to indicate likelihood of future doping. However it is very important to recognize that the doping BIAT does not measure doping, but attitudes to doping. It is possible that there are non-dopers with relatively positive attitudes to doping just as there may be dopers with relatively negative attitudes to doping.

In this study there were 16 individuals with positive *D*-scores (Figure 3). However IAT effects do not reveal whether an individual has a positive or negative attitude to a target in an absolute sense (13). It is inappropriate to equate a negative *D*-score with a negative attitude to doping. One reason for this is that it is not clear what psychological state corresponds to a zero *D-*score. Other reasons are inter-individually different subjective valences of focal and non-focal concepts (of the doping BIAT) and that the scale for reaction time-based psychological instruments is generally arbitrary [42]. An individual (or group mean) IAT *D-*score can only be interpreted relative to a comparison group.

Inter-individually different subjective valences of stimuli might be another source of bias connected with the test itself. For example the doping BIAT uses one picture with syringes to represent the concept of doping, while syringes themselves might already by negatively associated through prior personal experiences. Similarly, with regard to our choice of health food as the second category, indicators like a dish of cereals can be evaluated more or less positive likewise. According reservations can be addressed for example in the light of the associative-propositional evaluation (APE) model [17]. Within this model the concept of pattern activation refers to the idea that the relative fit between the pattern of preexisting associations in memory and the particular set of external input stimuli determines the activation of associations in memory. In the BIAT participants are instructed to decide whether presented stimuli either belong to the target category (here: doping) or to the attribute ‘like’ (or ‘dislike’ in the other test block). The second category (here: health food) throughout the test and the other attribute (‘dislike’) during the respective test block remain non-focal; participants are instructed to treat these stimuli as “anything else”. An associative focus [23], i.e. continuously linking syringes with doping so that the doping-related association pattern and not another one remains activated, is even emphasized as during all sorting trials the focal category labels remain visible on the screen. Of course this does not rule out the possibility that even better attitude measurements could be achieved with different picture stimuli or an alternative non-focal comparison category. Any given test has unique variance due to its specific test stimuli. All the pictorial doping BIAT’s stimuli were carefully pre-tested [25]. But maybe future studies can try to quantify differences that will most likely result from using alternative procedures [20].

42.6% of our bodybuilders’ urine samples contained doping substances. This prevalence is comparable to that reported by Delbeke and colleagues almost 20 years ago [27]. One very important conclusion from this earlier study was that one substance, the beta agonist clenbuterol, appeared in bodybuilders almost one year before it was first detected in competitive athletes from other sports. Competitive bodybuilding functions as a testing ground for innovations in doping (which may later spill over into Olympic sports). Our findings suggest that the number of bodybuilders who tend to experiment with drugs in an irritating irresponsible way is high. Excessively high T/E ratios (in 12 participants in this sample T/E was > 50), atypically high (>1,000 ng/ml) urinary concentrations of various steroids (e.g. drostanolone ~5,000 ng/ml) or their metabolites (e.g. 6-hydroxy-methandieonen ~2,000 ng/mL, 3’-hydroxy-stanozolol ~ 2,000 ng/mL) and suppression of the endogenous precursor of testosterone biosynthesis, luteinizing hormone (LH), are strong indicators of high-dose long-term abuse of multiple anabolic androgenic steroids. We found evidence of administration of vastly super-therapeutic dosages in several participants’ samples, and use of unapproved experimental drugs may be becoming more frequent. We detected the designer steroid methasterone in one urine sample; this drug and several other substances such as PPARδ receptor agonists, Growth Hormone Releasing Peptides (GHRPs) and Selective Androgene Receptor Modulators (SARMs), have been heavily advertised in the bodybuilding literature lately and are currently easily available from clandestine sources (and were recurrently confiscated by legality). In summary, our findings corroborate and extend the findings of case studies [43], administration of somewhat haphazard combinations of substances, complementary administration of substances, e.g. anti-oestrogens to treat side effects (gynecomastia) and use of stimulants to promote loss of body fat, are the norm rather than the exception for bodybuilders. Evidence suggesting use of depot preparations and the fairly high concentrations found in some samples do not suggest that our participants intended to avoid testing positive. This scenario contrasts sharply with that for Olympic sports.

This is the first study to use an indirect psychological test to assess attitudes to doping in competitive athletes (bodybuilders) whose doping status was confirmed with biochemical laboratory tests. It provided clear evidence – something previously lacking – that clean athletes’ attitudes to doping tend to be more negative than those of doped athletes. A recent review of relevant publications found only indirect evidence of the association between attitude to doping and doping behavior [7]. The pictorial doping BIAT identified almost three out of four doped athletes. This suggests that similar use of the pictorial doping BIAT in athletes from Olympic sport would probably produce a different picture regarding athletes’ doping attitudes than when data is obtained by direct enquiry in a questionnaire. Most of the extant questionnaire studies report floor effects, with the vast majority of respondents claiming to reject doping absolutely [44].

Last but not least it is important to note that our study does not provide evidence for the psychological theory of implicit cognition, i.e. social cognitive processes that possess features of automaticity [45]. What it does provide is evidence that response time latencies on a psychological test, which are thought to reflect learned associations between mental representations of semantic concepts (attributes and target concept), can be used to predict socially sensitive behavior at group level. These findings demonstrate an association between knowledge retrieved from our cognitive system and complex real life behavior (i.e. behavior measured outside artificial research settings). There have been few reports of such associations so this result is of some importance [46].

### Perspectives

This is the first study to provide evidence of the external validity of an indirect test of athletes’ attitudes to doping. We suggest that indirect tests of attitude to doping could be used as part of a portfolio of outcome measures [47,48] in evaluations of doping prevention programs. In our view there is an urgent need for robust evaluation of anti-doping intervention; perhaps because the social desirability bias makes it difficult to elicit honest expression of attitudes to doping directly from athletes, very few anti-doping interventions are evaluated at all (e.g. [49]) and none has used anything other than a questionnaire to assess psychological effects. As well as demonstrating that it is possible to predict with reasonable accuracy whether an athlete is a doper on the basis of a pictorial doping BIAT *D-*score, this study can be seen as an attempt to promote the very necessary shift from output-based (e.g. how many anti-doping interventions were delivered, reaching how many athletes) to outcome-based evaluations of doping prevention programs in sport (i.e. what difference did they make, [50]).

## Competing interests

The authors declare that they have no competing interests.

## Authors’ contributions

R.B. designed the study. W.W. organized the data collections, D.T. supervised the biochemical analyses of the urine samples. W.W. conducted the statistical calculations. All authors discussed the results, R.B. wrote the first draft of the manuscript. All three authors then jointly worked on finalizing the manuscript. All three authors read and approved the final manuscript.

## Supplementary Material

Additional file 1: Table S1 BIAT D-scores and results of biochemical testing for all bodybuilders. Click here for file
