# Supplementary material for: Using response-time latencies to measure athletes’ doping attitudes: the brief implicit attitude test identifies substance abuse in bodybuilders
Source: Subst Abuse Treat Prev Policy. 2014 Sep 10;9:36. doi: 10.1186/1747-597X-9-36 (PMC4163696; doi:10.1186/1747-597X-9-36)
Supplement: Additional file 1: Table S1 — BIAT D-scores and results of biochemical testing for all bodybuilders. [file 1747-597X-9-36-S1.pdf]

Supplementary Material, Table.
BIAT *D*-scores and results of biochemical testing for all bodybuilders

Participant [Code]	BIAT D score	pH	Density	T/E	Testosterone [ng/mol]	Substances according to WADA categorization S1	S2	S4	S6	S8
2000	-1.22	-	-	-	-	-	-	-	-	-
105	-1.14	-	-	-	-	-	-	-	-	-
131	-1.13	-	-	-	-	-	-	-	-	-
110	-1.08	-	-	-	-	-	-	-	-	-
20	-1.05	5.8	1.022	48.6	951	Boldenone < MRPL<br>Drostanolone < MRPL<br>Nandrolone				
122	-1.03	-	-	-	-	-	-	-	-	-
2001	-1.02	-	-	-	-	-	-	-	-	-
117	-0.99	-	-	-	-	-	-	-	-	-
108	-0.98	-	-	-	-	-	-	-	-	-
31	-0.97	-	-	-	-	-	-	-	-	-
1120	-0.90					-	-	-	-	-
13	-0.85	5.3	1.026	48.6	299	Boldenone<br>Drostanolone < MRPL<br>Nandrolone<br>Trenbolone < MRPL		Tamoxifene		cTHC ca. 1700 ng/ml
114	-0.84	-	-	-	-	-	-	-	-	-
1116	-0.84	-	-	-	-	-	-	-	-	-
17	-0.79	6.3	1.025	90.6	472	Boldenone<br>Drostanolone<br>Metenolone <		Anastrozol<br>Tamoxifene		

						MRPL<br>Nandrolone<br>Trenbolone				
107	-0.79	-	-	-	-	-	-	-	-	-
104	-0.78	-	-	-	-	-	-	-	-	-
9	-0.68	-	-	-	-	-	-	-	-	-
15	-0.66	6.9	1.019	132	484	Boldenone<br>DHCMT (nur LZM)<br>Nandrolone<br>Trenbolone		Anastrozol<br>Tamoxifene		
121	-0.63	-	-	-	-	-	-	-	-	-
1123	-0.62	-	-	-	-	-	-	-	-	-
119	-0.61	-	-	-	-	-	-	-	-	-
1112	-0.59	-	-	-	-	-	-	-	-	-
05	-0.56	5.0	1.023	16.8	125	Boldenone<br>19-NA ca. 1 ng/ml				
25	-0.55	-	-	-	-	-	-	-	-	-
26	-0.55	5.7	1.007	6.7	38.8				MDMA / MDA	cTHC ca. 40 ng/ml
01	-0.55	6.0	1.022	7.1	3.7	Methandienone<br>19-NA ca. 2 ng/ml<br>Boldenone < MRPL				
120	-0.50	-	-	-	-	-	-	-	-	-
10	-0.48	5.9	1.025	86.2	1528	Boldenone<br>Nandrolone<br>Trenbolone				
115	-0.47	-	-	-	-	-	-	-	-	-
14	-0.40	5.8	1.023	2.2	24.6	Stanozolol			Methylhexanamin	cTHC ca.

									e	4800 ng/ml
28	-0.38	-	-	-	-	-	-	-	-	-
1117	-0.38	-	-	-	-	-	-	-	-	-
21	-0.32	5.5	1.014	50	47.5	Clenbuterol<br>19-NA ca. 2 ng/ml<br>Trenbolone				
02	-0.23	5.7	1.018	7.1	13.9	Boldenone<br>Drostanolone<br>Metenolone<br>Nandrolone<br>Stanozolol<br>Trenbolone	hCG	Tamoxifene		
27	-0.26	-	-	-	-	-	-	-	-	-
101	-0.24	-	-	-	-	-	-	-	-	-
07	-0.21	6.6	1.021	37.7	102	Boldenone ca. MRPL<br>Methandienone<br>19-NA ca. 1 ng/ml				
03	-0.09	6.1	1.015	53.3	278				Sibutramine < MRPL	
12	-0.08	6.6	1.028	14.2	75.4	Boldenone<br>Nandrolone<br>Methandienone				
1115	-0.03	-	-	-	-	-	-	-	-	-
123	-0.02	-	-	-	-	-	-	-	-	-
06	-0.02	6.6	1.014	9.3	3.3	Trenbolone < MRPL<br>19-NA ca. 6 ng/ml				
24	-0.01	6.2	1.023	87.8	464	Drostanolone		Tamoxifene	Ephedrine ca. 24	

						Trenbolone < MRPL		< MRPL	µg/ml	
1113	0.00	-	-	-	-	-	-	-	-	-
118	0.00	-	-	-	-	-	-	-	-	-
116	0.01	-	-	-	-	-	-	-	-	-
11	0.03	5.9	1.026	185	1317	Boldenone < MRPL<br>Drostanolone < MRPL<br>Nandrolone				
32	0.06	-	-	-	-	-	-	-	-	-
1111	0.09	-	-	-	-	-	-	-	-	-
30	0.12	5.7	1.019	7.3	84.1	Boldenone < MRPL				
19	0.15	5.8	1.019	6.4	46.5	Boldenone<br>Nandrolone<br>Trenbolone				
16	0.16	6.0	1.014	51.6	72.2	Boldenone<br>Drostanolone < MRPL<br>Methandienone<br>Methasterone-M ca. MRPL<br>19-NA ca. 5 ng/ml<br>Oxymetholone (nur LZM)<br>Stanozolol				
04	0.20	5.2	1.019	22.4	12	Boldenone<br>Stanozolol		Tamoxifene	Methylhexanamine	
29	0.23	5.8	1.023	77.8	322	Boldenone<br>Drostanolone <				

						MRPL 19-NA ca. 4 ng/ml				
22	0.23	5.6	1.022	58.2	639	Boldenone			Amphetamine	
18	0.47	5.4	1.029	13.1	19.1	Boldenone Nandrolone				
109	0.69	-	-	-	-	-	-	-	-	-
106	0.77	-	-	-	-	-	-	-	-	-
23	0.91	6.1	1.021	73.1	286	19-NA ca. 2 ng/ml				
08	1.02	5.7	1.018	85.8	191	Drostanolone Methandienone Nandrolone		Tamoxifene		
